# Dynamic Patterns of Circulating Seasonal and Pandemic A(H1N1)pdm09 Influenza Viruses From 2007–2010 in and around Delhi, India

**DOI:** 10.1371/journal.pone.0029129

**Published:** 2012-01-03

**Authors:** Shobha Broor, Anand Krishnan, Dipanjan S. Roy, Shivram Dhakad, Samander Kaushik, Muneer A. Mir, Yashpal Singh, Ann Moen, Mandeep Chadha, Akhilesh C. Mishra, Renu B. Lal

**Affiliations:** 1 All India Institute of Medical Sciences, Delhi, India; 2 Influenza Division, Centers for Disease Control and Prevention, Atlanta, Georgia, United States of America; 3 National Institute of Virology, Pune, India; Massey University, New Zealand

## Abstract

Influenza surveillance was carried out in a subset of patients with influenza-like illness (ILI) presenting at an Employee Health Clinic (EHS) at All India Institute of Medical Sciences (AIIMS), New Delhi (urban) and pediatric out patients department of civil hospital at Ballabhgarh (peri-urban), under the Comprehensive Rural Health Services Project (CRHSP) of AIIMS, in Delhi region from January 2007 to December 2010. Of the 3264 samples tested, 541 (17%) were positive for influenza viruses, of which 221 (41%) were pandemic Influenza A(H1N1)pdm09, 168 (31%) were seasonal influenza A, and 152 (28%) were influenza B. While the Influenza viruses were detected year-round, their types/subtypes varied remarkably. While there was an equal distribution of seasonal A(H1N1) and influenza B in 2007, predominance of influenza B was observed in 2008. At the beginning of 2009, circulation of influenza A(H3N2) viruses was observed, followed later by emergence of Influenza A(H1N1)pdm09 with co-circulation of influenza B viruses. Influenza B was dominant subtype in early 2010, with second wave of Influenza A(H1N1)pdm09 in August-September, 2010. With the exception of pandemic H1N1 emergence in 2009, the peaks of influenza activity coincided primarily with monsoon season, followed by minor peak in winter at both urban and rural sites. Age group analysis of influenza positivity revealed that the percent positivity of Influenza A(H1N1)pdm09 influenza virus was highest in >5–18 years age groups (OR 2.5; CI = 1.2–5.0; *p* = 0.009) when compared to seasonal influenza. Phylogenetic analysis of Influenza A(H1N1)pdm09 from urban and rural sites did not reveal any major divergence from other Indian strains or viruses circulating worldwide. Continued surveillance globally will help define regional differences in influenza seasonality, as well as, to determine optimal periods to implement influenza vaccination programs among priority populations.

## Introduction

Influenza is a widespread viral infection and a major cause of morbidity and mortality worldwide [1], [2]. The WHO Global Influenza Surveillance Network has greatly contributed to the knowledge about circulating influenza viruses, including emergence of novel strains [3], [4]. Improved understanding of temporal and geographic circulation of influenza viruses and the impact of influenza among populations living in tropical and subtropical regions is essential for the development of influenza prevention and control strategies for those areas [1], [5], [6]. The threat of an avian influenza virus (H5N1) pandemic and the emergence of 2009 pandemic Influenza, represented major stimuli for advances in knowledge about influenza in many countries [1], [7].

The seasonality of influenza in the tropical regions varies considerably from that in temperate regions [1], [5], [6]. In temperate regions of the Northern and Southern Hemispheres, annual winter epidemics are associated with excess deaths from influenza and pneumonia [2], [8]. Influenza activity in tropical countries usually occurs year round with peaks coinciding in some countries with rainy season, whereas other countries only have an influenza peak in the rainy season without significant activity during the rest of the year [5], [6], [9], [10]. Recent studies from Bangladesh, Cambodia, India, Laos, Myanmar, Singapore, Thailand, and Vietnam have further shown the importance of burden of influenza-related illness in the Asian region [5], [8]–[15]. Thus, studying both the incidence and seasonality of influenza is crucial for development of effective regional preventive strategies, including identification of virus strains for vaccine selection.

Although influenza is recognized as an important cause of acute respiratory illness [2], [6], [8], [12], little is known about the prevalence and burden of influenza in India. A systematic laboratory-based surveillance network of influenza viruses was established that includes sentinel surveillance sites geographically distributed in northern, central, southern, and eastern India [15]. The surveillance network is generating data to better understand the circulating subtypes and seasonality in different geographic regions in India. In the current report, we summarize surveillance data of Influenza-like illness(ILI) presenting for care in urban and peri-urban sites in and around Delhi for the period 2007–2010, which includes surveillance during the Influenza pandemic and post-pandemic periods.

## Results

### Influenza positivity and seasonality in Sentinel surveillance site in Northern India

**Figure 1:**
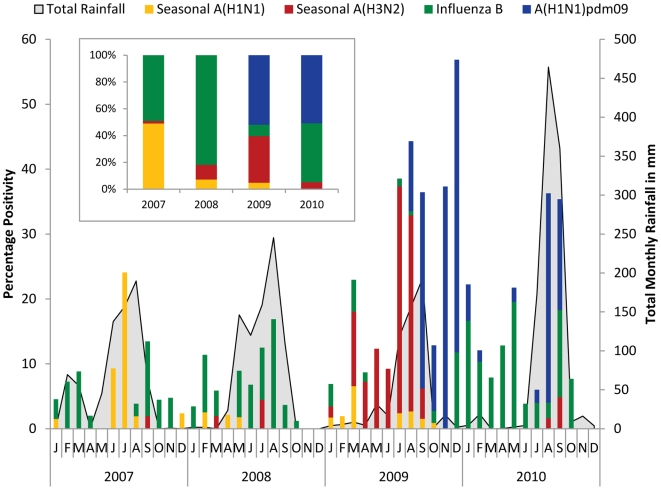
Monthly trends and seasonality of influenza viruses in Delhi. The left axis shows the percent monthly distribution of seasonal influenza (Yellow bar A(H1N1); red bar A(H3N2); and green bar representing Influenza B) and pandemic A(H1N1)pdm09 (Blue line) with total monthly rainfall (grey shaded) is shown on right axis for each of the years. The inset shows overall distribution of Influenza types and subtypes for years 2007–2010.

A total of 3264 specimens from years 2007 (n = 510), 2008 (n = 822), 2009 (n = 1071), and 2010 (n = 661) were tested for Influenza either by virus isolation or by real-time RT-PCR (since April 2009). Of these, 541/3264 (17%) were positive for influenza viruses (Table 1). Influenza positivity was lower in 2007 (55/710; 8%) and 2008 (55/822; 7%) followed by a marked increase in influenza positivity in 2009 (315/1071; 29%), primarily due to emergence of Influenza A(H1N1)pdm09 in August of 2009 (Table 1). More moderate rates were seen in 2010 (116/661; 17%), with circulation of Influenza B in first half and a second wave of Influenza A(H1N1)pdm09 in August-September of 2010 (Fig. 1).

**Table 1:** Surveillance for Influenza-like-Illness in and around Delhi, North India, 2007–2010.

Year	# Tested	Influenza positive		Influenza B		Seasonal Influenza A				Influenza A(H1N1)pdm09	
						H3N2		H1N1			
		n	%	N	%*	n	%*	n	%*	n	%*
**2007**	710	55	8	27	49	1	2	27	49	NA	NA
**2008**	822	55	7	46	84	6	11	3	5	NA	NA
**2009**	1071	315	29	28	9	110	35	15	5	162	51
**2010**	661	116	17	51	44	6	5	0	0	59	51
**Total**	**3264**	**541**	**17**	**152**	**28**	**123**	**23**	**45**	**8**	**221**	**41**

*Denominator for the percentage is # influenza positive for that year.

Analysis of various meteorological factors revealed that the peak of influenza positivity for each year from 2007 to 2010 coincided with peak of total rainfall during the monsoon season (July-August) in Delhi area (Fig. 1). Further, a statistically significant correlation (r = 0.4; *p* = 0.005) was observed between influenza positivity and rainfall (data not shown).

### Influenza types and subtypes in Northern India

While the Influenza viruses were detected year-round, the types/subtype varied remarkably. The month wise percentage distribution of influenza viruses was plotted over the total number of samples positive for influenza viruses in the respective year (Fig. 1). There was equal distribution of seasonal influenza A(H1N1) and influenza B in 2007 and predominance of influenza B viruses in 2008. In 2009, seasonal A(H1N1) viruses were completely replaced with Influenza A(H1N1)pdm09 viruses. Influenza A(H3N2) viruses circulated at low levels in 2008, their activity increased just before emergence of Influenza A(H1N1)pdm09 in August 2009. In 2010, there was co-circulation of Influenza B, A(H3N2) and influenza A(H1N1)pdm09 viruses. The Influenza A(H1N1)pdm09 demonstrated very discrete pattern with a bimodal peaks appearing in September and December of 2009, and a single peak in 2010 (Fig. 1). Overall, Influenza A (H1N1) and Influenza B accounted for almost equal distribution in 2007, with predominance of influenza B in 2008 (>80%) (Fig. 1 inset). Influenza A(H1N1)pdm09 accounted for >50% infections in both 2009 and 2010, whereas A(H3N2) accounted for >35% infections in 2009 and influenza B accounted for >44% infections in 2010 (Table 1, Fig. 1 inset).

### Influenza among urban and peri-urban sites in Northern India

Since sentinel surveillance in Delhi represents a diverse population, we compared the prevalence and seasonality of influenza in urban (EHS clinic at a tertiary care hospital at AIIMS, New Delhi; n = 1816; ages 1 mo to >50 yrs) and peri-urban (Pediatric OPD, a secondary level hospital at Ballabhgarh, Haryana; n = 1448; ages 1 mo to <18 yrs) settings (Table 2). Influenza positivity was lower in 2007–08 in both urban (6–7%) and peri-urban (7–9%) areas and higher in both these areas (urban 23–35%; peri-urban 13–19%) in 2009–2010 (Table 2). The reasons for lower influenza positivity in 2007–2008 could be, in part, due to detection methodologies, as all samples till March 2009 were tested by virus isolation methods, whereas real-time RT-PCR testing was implemented thereafter.

**Table 2:** Influenza positivity among Urban and peri-urban Population in and around Delhi, North India, 2007–2010.

Year		# tested	# Influenza +ve		Influenza B		SeasonalInfluenza A				InfluenzaA(H1N1)pdm09	
							H3N2		H1N1			
			n	%	n	%*	n	%*	n	%*	n	%*
2007	Urban	417	28	7	11	39	1	4	16	57	NA	NA
	Peri-urban	293	27	9	16	59	0	0	11	41	NA	NA
2008	Urban	410	26	6	22	85	3	11	1	4	NA	NA
	Peri-urban	412	29	7	24	83	3	10	2	7	NA	NA
2009	Urban	675	238	35	26	11	55	23	9	4	**148** Ψ	62
	Peri-urban	396	77	21	2	3	**55** €	71	6	8	14	18
2010	Urban	314	72	23	24	33	6	8	0	0	**42** Ψ	58
	Peri-urban	347	44	13	27	61	0	0	0	0	17	39
Total	Urban	1816	364	20	83	23	65	18	26	7	190	52
	Peri-urban	1448	177	12	69	39	58	33	19	11	31	17

*Denominator for the percentage is # influenza positive for that year.

€ *p*<0.01 (highly significant for Influenza A (H3N2) in 2009, OR = 1.8, CI – 1.2–2.7)for peri-urban area.

Ψ *p*<0.001 (highly significant for pandemic Influenza A(H1N1)pdm09 in 2009, OR = 7.7, CI – 4.2–14) and 2010 (OR = 3.0, CI – 1.6–5.6) for urban areas.

### Seasonality and circulating strains in urban and peri-urban sites in Northern India

**Figure 2:**
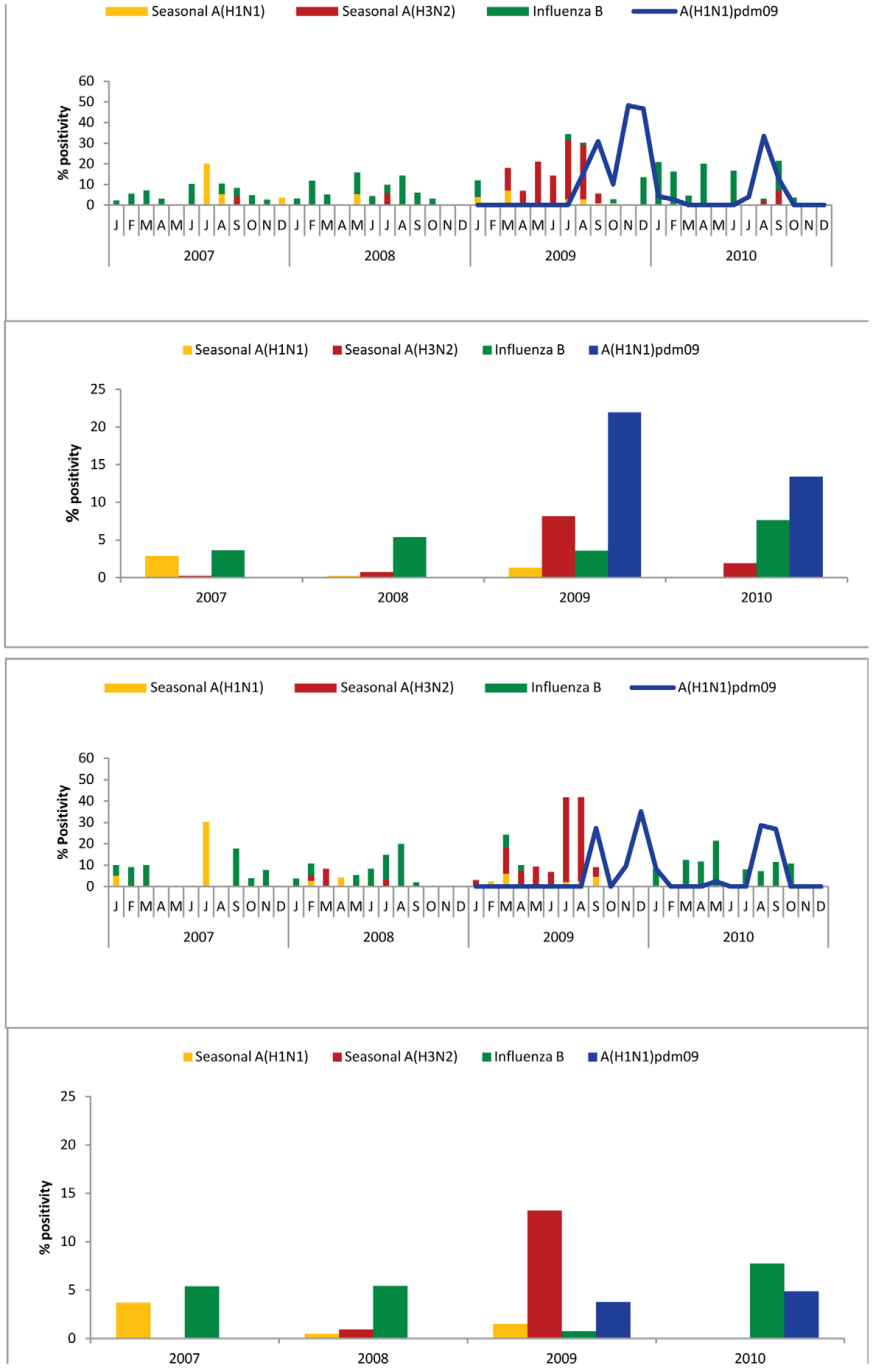
Monthly trends and seasonality of circulating seasonal and pandemic influenza viruses in urban (Panel A) and peri-urban (Panel B) area from 2007–2010. The left axis shows monthly percent positivity. The overall % positivity of types and subtypes is shown as bar graph for urban (Top) and peri-urban (bottom) area from 2007 to 2010.

To understand the seasonality and trend of circulating influenza strains, the month wise percentage distribution of influenza viruses was plotted over the total number of samples positive for influenza in the respective years from 2007–2010 for urban (Fig. 2A) and peri-urban sites (Fig. 2B). Both urban and peri-urban sites revealed almost year round circulation with a major peak in rainy season (July–August), and a minor peak in winter-spring season (December–February). Likewise, timing of emergence of Influenza A(H1N1)pdm09 also was similar in urban (Fig. 2A) and peri-urban (Fig. 2B) sites, with bimodal peaks in September and December 2009, and a single major peak in August–September of 2010. Whereas no difference in type or subtype distribution was discernable between urban and peri-urban sites in 2007 and 2008, marked differences were observed during pre-pandemic and pandemic periods at these sites. While A(H3N2) accounted for <25% of strains in the urban site, a surge in A(H3N2) (>70%) was observed prior to emergence of pandemic H1N1 in the peri-urban area (Table 2; Fig. 2B). Whereas Influenza A(H1N1)pdm09 accounted for >60% of circulating strains at the urban site, less than 20% positivity was observed at peri-urban site (Fig. 2A; Table 2). Positivity for Influenza A(H1N1)pdm09 was higher (p<0.001) in the urban area when compared with peri-urban in both 2009 and 2010 (Table 2). In addition, we observed persistence of influenza B during post pandemic period in 2010 in the peri-urban area (Fig. 2B) compared with the urban area (Fig. 2A). There were some differences in the timing of peak of influenza B circulation, it peaked in January and April 2010 in urban areas (Fig. 2A), but not until May 2010 in peri-urban area (Fig. 2B).

**Figure 3:**
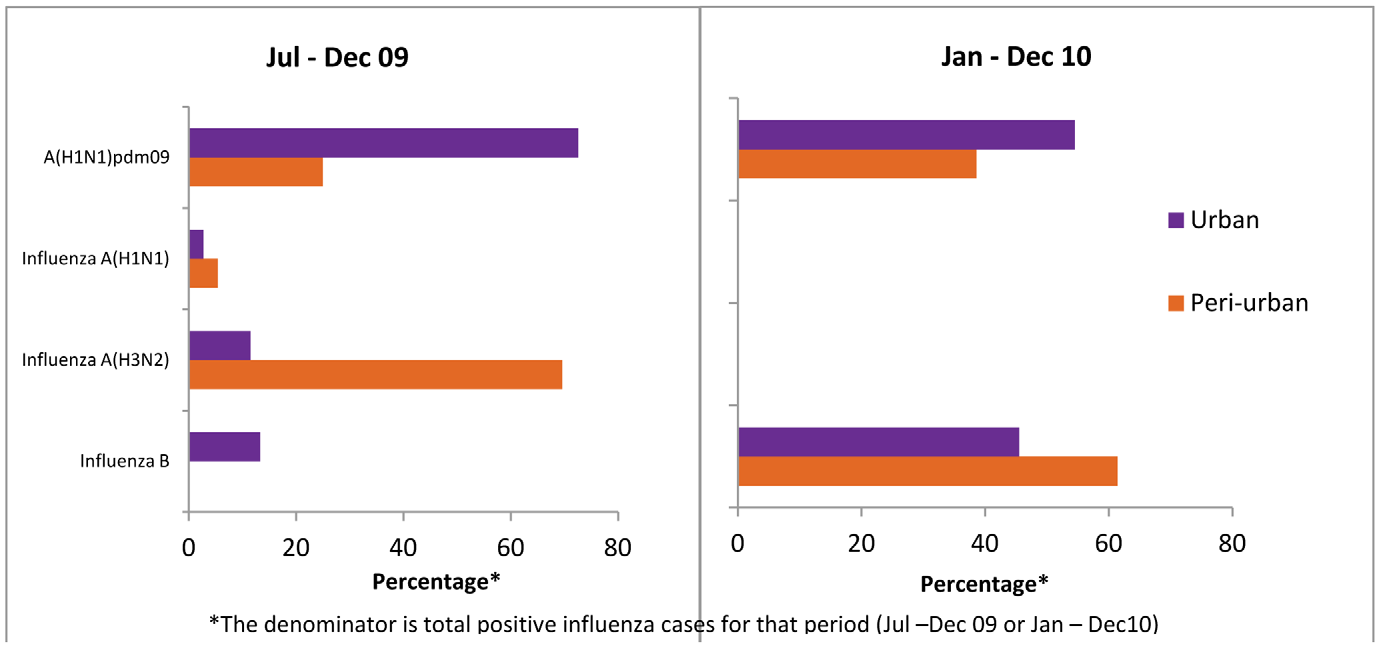
Comparative analysis of circulating influenza viruses (Influenza A and B) among children <18 years of age in urban (purple) and peri-urban (orange) setting from July-December 2009 (left panel) and January-December 2010 (right panel).

Since the peri-urban site data represents only children, we also examined the distribution of circulating strains in children <18 years of age from July 2009–December 2010 when real time PCR data was available for both sites. Overall, the seasonality of circulating strains at the urban site among children <18 years was fairly comparable to that seen for the entire urban study population. The percentage of Influenza A(H1N1)pdm09 viruses was significantly higher at the urban site for 2009 and 2010, among children <18 years whereas the peri-urban site had a higher prevalence of influenza A(H3N2) in 2009 and Influenza B in 2010 (Fig. 3).

### Age group for Influenza positivity in urban population

Analysis of the data from the pre-pandemic period from January 2007 to July 2009 (not shown) or that of a cumulative analysis of the data collected from 2007–2010 (n = 1816) revealed that age groups >5–18 and >18–25 years old had the highest rates of influenza positivity ranging from 11–15% during pre-pandemic phase and 27–30% during entire study period. Further analysis from July 2009 (at the time of emergence of pandemic influenza in Delhi) to December 2010, also revealed that the highest influenza positivity was observed in the >5–18 years old age group (112/266; 42%), followed by the >18–25 years old group (42/108; 39%). More importantly Influenza A(H1N1)pdm09 positivity was 29% and 27.8% respectively among children between >5–18 (78/112; OR 2.5; CI = 1.2–5.0; *p* = .009) and >18–25 years of age (30/42; OR 2.7; CI = 1.13–6.5; p = .02) (Table 3) when compared to >35 years age group. Further, the percentage positivity for Influenza A(H1N1)pdm09 was higher (19.8%–29.3%) in all age group except >35 years age group than seasonal influenza positivity (8.6%–11.8%) (Table 3).

**Table 3:** Distribution of ILI cases, seasonal and Influenza A(H1N1)pdm09 positives by age groups in urban population from July 2009–December 2010.

Age in yrs	# Tested	Influenza +ve		Seasonal Influenza +ve		Influenza A(H1N1)pdm09 +ve		Odds ratio (Prevalence Ratio)	95% CI	P value(Pearson's Chi squared test)
		n	%	n	%	n	%			
0–5	81	23	28.0	7	8.6	16	19.8	2.5	0.87–7.1	0.09
>5–18	266	112	42.0	34	12.8	78	29.3	2.5	1.2–5.0	**0.009**
>18– 25	108	42	39.0	12	11.1	30	27.8	2.7	1.13–6.5	**0.02**
>25–35	194	66	34.0	23	11.8	43	22.2	2.0	0.95–4.3	0.06
>35	214	48	23.0	25	11.7	23	10.7	Baseline		
Total	863	291	34.0	101	11.7	190	22.0			

### Phylogenetic analysis of Influenza A(H1N1)pdm09 sequences

**Figure 4:**
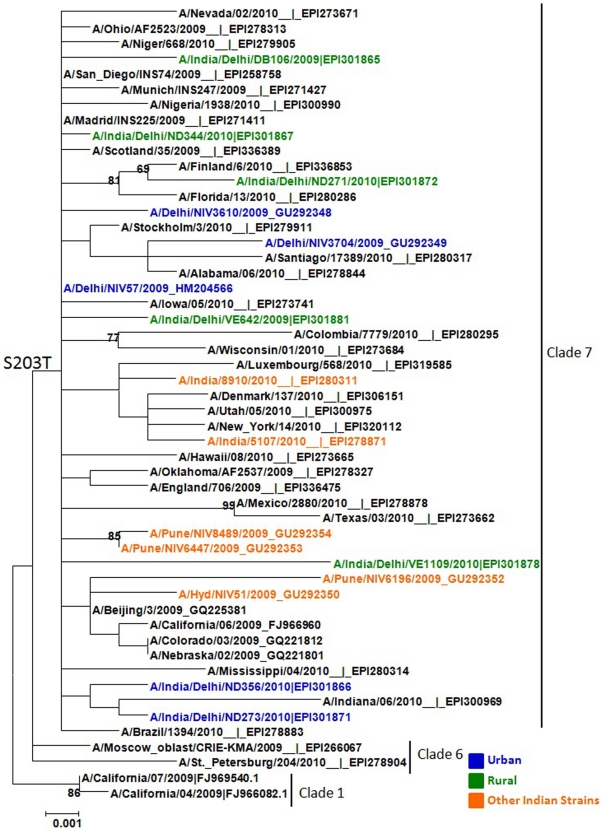
Phylogenetic analysis of HA gene of Influenza A(H1N1)pdm09 strains isolated from Urban (blue) and peri-urban (green) sites from Delhi region, North India in Aug 2009 or 2010. The representative strains from 2009/A/California(H1N1) strain and from other parts of the world and India (Orange) were used to generate the phylogenetic tree. The characteristic amino acids unique to clade 7 (S203T) is shown at the root of the branch.

Phylogenetic analysis of randomly selected ten Influenza A(H1N1)pdm09 isolates from urban or peri-urban (Fig. 4) areas revealed that these strains clustered with clade 7 from other parts of world, as well with other sequences from India. Clade 7 viruses are characterized by S203T mutations in the HA1 gene of Influenza A(H1N1)pdm09. No specific mutations could be identified between viral sequences, suggesting that the Influenza A(H1N1)pdm09 circulating strains were comparable in urban or peri-urban area.

## Discussion

Influenza surveillance indicates that influenza viruses circulate year-round in the Delhi (North India) area and that influenza contributes significantly to the number of ILI patients seeking care in government facilities in urban and peri-urban areas of Delhi, especially during the rainy and winter seasons. Although patients with ILI were seen throughout the year, there were distinct seasonal peaks in influenza virus isolations from ILI cases in July-August which coincided with peak rainfall in most years. Overall, both influenza A and B viruses co-circulated throughout the surveillance period. Seasonal influenza A(H1N1) and A(H3N2) viruses, however, predominated during distinct periods with little overlap, i.e. seasonal A(H1N1) was the predominant circulating virus in 2007, whereas A(H3N2) showed some circulation throughout the study period, with a marked increase in circulation in the first half of 2009. Our observation of rapid displacement of circulating A(H3N2) by pandemic virus during the initial phase of 2009 pandemic has also been observed in other parts of the world [7], [14]. The dynamic patterns and displacement of one subtype with another may partly be explained by limited immunological cross-reactivity between influenza subtypes. Pandemic influenza A(H1N1)pdm09 did not show typical seasonality in 2009 with bimodal peaks observed in September and December 2009, but a distinct peak in rainy season (August-September) was seen in 2010 in Delhi. We observed a consistent pattern of peak influenza activity in both urban and peri-urban surveillance sites, with some differences in circulating strains in urban and peri-urban areas.

The temporal peaks in influenza virus circulation coincided with rainy and winter seasons in and around Delhi. Our findings of seasonal peaks of influenza activity in India are consistent with data reported from surrounding countries in the region, where peaks of influenza activities coincide with rainy seasons [9]–[13]. In some tropical regions, there is high background influenza activity throughout the year with distinct peaks appearing during monsoon or cooler months [5], [6]. While the exact mechanisms leading to variation in influenza seasonality are not clear, attempts to correlate fluctuation in meteorological variables have shown relationship with influenza positivity during the rainy season in the tropics [16], [17]. Multi-site influenza surveillance from different geographic regions in India has also revealed a positive correlation between the rainy season and rates of influenza virus isolation [15]. The seasonality of influenza in Delhi area has policy implications such as vaccination timing and the use of northern vs southern hemisphere vaccine formulations in this region.

A strong surveillance system which encompassed diverse settings enabled us to monitor the emergence of Influenza A(H1N1)pdm09 in and around Delhi, North India. During the initial pandemic phase, we observed a higher prevalence of seasonal influenza A/H3N2 than Influenza A(H1N1)pdm09. This observation is different from some cities in Mexico and United States, where >90% of the positive samples were due to 2009(H1N1) influenza during the first wave of pandemic [1], [18]. The trend in Delhi changed in September, when Influenza A(H1N1)pdm09 became predominant, and a distinct bimodal pattern of peak activity was observed in September and December 2009, possibly due to colder weather where influenza viruses transmit more readily [5], [6]. These observations are similar to situations in other parts of the world where Influenza A(H1N1)pdm09 either completely replaced seasonal influenza [14], [18]–[20] or co-circulated with seasonal influenza viruses [1], [7]. During previous pandemics, the novel virus subtype has replaced previously circulating viruses [7]. For example, H1N1 was replaced by H2N2 in 1957, subsequently H3N2 emerged in 1968, and H1N1 reemerged in 1977 [21]. The latter two have co-circulated since then, although in one season only one or the other tends to dominate [5], [7]. Now the Influenza A(H1N1)pdm09 virus has replaced the seasonal influenza A(H1N1) virus circulating in preceding years.

We observed influenza B circulation in the early part of 2010, followed by second wave of Influenza A(H1N1)pdm09 in August 2010, similar to what is observed in present times in most countries around the globe [22]. While the exact mechanism causing such a discrete peak in August 2010 is not clear, molecular studies suggest that large populations in the tropics can serve as reservoirs of influenza infection throughout the year, with reseeding of drifted viruses possibly leading to outbreaks [4], [23]. Whether such a drift in Influenza A(H1N1)pdm09 could account for the peak remains to be determined, though to date minimal divergence among the limited sequences of Influenza A(H1N1)pdm09 makes this explanation unlikely [24]–[27]. Alternatively, some environmental factors may play a role in virus survival and it is plausible that drastic changes in meteorological parameters, such as lowering of vapor pressure [17], [28], may account for a sharp peak of Influenza A(H1N1)pdm09 in August 2010.

Another important aspect of our study is the comparative analysis of influenza seasonality and circulating virus strains in urban and peri-urban settings. Little is known about how demographic, nutritional and other environmental factors impact circulating influenza viruses, although vaccine studies have shown considerable differences in humoral and cell mediated immune responses to influenza vaccination among semi-urban and rural school children in Gabon [29]. In the present study, peaks of influenza circulation were comparable in urban and peri-urban areas, but circulating strains varied somewhat in some years. For instance, a higher proportion of circulating A(H3N2) viruses was observed in the peri-urban site in July-August 2009, when compared to urban site. Whether a new antigenic variant of H3N2 had emerged, to which there was only partial immunity in the peri-urban site, remains to be determined. Studies are underway to better understand the evolution of H3N2 viruses from these surveillance studies.

Age group analysis revealed that the highest rate of influenza A(H1N1)pdm09 positivity was observed among children in the age group >5–18 yrs, which could be due to high exposure rates among school aged-children, who have highest numbers of contacts among all age groups, and therefore, appear to be at the leading edge of pandemic [30], [31]. The attack rates of Influenza A(H1N1)pdm09 have varied among various age groups in different parts of the world, however, most have found children to represent the highest risk group [31]–[33]. Recent modeling study has suggested that while school-age children typically experience the highest attack rates during early pandemic phase, the burden likely shifts to adults during the subsequent season [34]. It remains to be seen if Influenza A(H1N1)pdm09 strains of influenza may shift to adults in the next wave.

Phylogenetic analysis of HA1sequences revealed that all belonged to clade 7 with minimal diversity in sequences whether derived from urban or peri-urban area, and all had signature sequence S203T in the HA1 subunit [25]–[27], [35]. Our data are in concordance with published data where clade 7 of Influenza A(H1N1)pdm09 viruses remain the predominant circulating strains globally [25], [26].

Taken together, our data provides evidence that the seasonality of influenza in Delhi area is related to rainfall, although the Influenza A(H1N1)pdm09 emerged after the rains stopped in 2009. Further, we demonstrate that seasonal influenza types and subtypes change constantly and unpredictably over time. In summary, continued surveillance globally will help to better define seasonal patterns in the circulation of influenza A and B viruses, regional differences in influenza seasonality, as well as to determine optimal periods to implement influenza vaccination programs among priority populations.

## Materials and Methods

### Case Definition of Influenza like illness (ILI)

A person presenting with sudden onset of fever >38° C or history of sudden onset of fever in the recent past (less than 3 days), with cough or sore throat.

### Study Population

ILI surveillance was established at AIIMS in December, 2004, and is conducted at two sentinel sites [19]: Employee's Health Clinic (EHS) at AIIMS, New Delhi, which is attended by all AIIMS employees and their families (urban) (includes all strata with low, middle and high income levels), and at the pediatric Outpatient department (OPD) at the Comprehensive Rural Health Services Project (CRHSP) at Ballabhgarh (peri-urban) (with low to middle income strata). This outpatients department is visited by the peri-urban population of Ballabhgarh as well as by the rural population in this block. Study physicians visit AIIMS clinic four times a week and Ballabhgarh clinic twice a week. At least 5–10 nasopharyngeal samples are collected from patients meeting the ILI case definition on first come first served basis each week. The average specimen collection has been fairly consistent throughout the study period with median number of ILI specimens collected per month were 61 (range 42–83) in 2007, 66 (range 51–112) in 2008, 74 (range 52–153) in 2009 and 48 (range 26–124) in 2010.

### Ethical clearance

The study was approved by Institutional Review Board of AIIMS and by the Indian Health Ministry's Screening Committee. Informed consent was taken from all patients participating in the surveillance protocol.

### Laboratory Diagnosis

Combined throat and nasal swabs from ILI patients were collected and transported to the virology laboratory as described previously [19], [35]. From 2007 till March 2009, all samples from sentinel surveillance sites were tested for influenza viruses (A and B) by virus isolation in MDCK cells [15]. Since April 2009, all the samples were tested by real-time RT-PCR for the detection of influenza viruses and influenza A virus positive samples were further subtyped as described [35], [36]. A confirmed case was defined as a patient meeting the ILI case definition and either yielding an isolate of influenza in MDCK cell line or positive for influenza by RT-PCR.

### Sequencing and Phylogenetic Analysis

The haemagglutinin 1 (HA-1) gene was sequenced by the dideoxynucleotide chain termination method as described previously [35]. The GISAID accession numbers are EPI247057-58, EPI259029, EPI301865-67, EPI301871-72, EPI301878, EPI301881.

### Statistical Analysis

Pearson's correlation coefficient (r) was used to calculate the linear relationship between rainfall aggregated for the month and influenza positivity. The aggregate rainfall data was collected from Indian Meteorological Department (http://www.imd.gov.in/section/hydro/distrainfall/webrain/delhi/delhi.txt) for the study period. Odds ratio with Pearson's Chi Squared test was used due to multiple attribute dataset (qualitative) to show the significant age group. Statistical analysis was done using STATA 11 software.
